# Sample size re-assessment leading to a raised sample size does not inflate type I error rate under mild conditions

**DOI:** 10.1186/1471-2288-13-94

**Published:** 2013-07-19

**Authors:** Per Broberg

**Affiliations:** 1 Department of Oncology and Cancer Epidemiology, Clinical Sciences, Lund University and Skåne University Hospital, 221 85 Lund, Sweden

**Keywords:** Adaptive trial design, Type I error, Conditional power

## Abstract

**Background:**

One major concern with adaptive designs, such as the sample size adjustable designs, has been the fear of inflating the type I error rate. In (Stat Med 23:1023-1038, 2004) it is however proven that when observations follow a normal distribution and the interim result show promise, meaning that the conditional power exceeds 50%, type I error rate is protected. This bound and the distributional assumptions may seem to impose undesirable restrictions on the use of these designs. In (Stat Med 30:3267-3284, 2011) the possibility of going below 50% is explored and a region that permits an increased sample size without inflation is defined in terms of the conditional power at the interim.

**Methods:**

A criterion which is implicit in (Stat Med 30:3267-3284, 2011) is derived by elementary methods and expressed in terms of the test statistic at the interim to simplify practical use. Mathematical and computational details concerning this criterion are exhibited.

**Results:**

Under very general conditions the type I error rate is preserved under sample size adjustable schemes that permit a raise. The main result states that for normally distributed observations raising the sample size when the result looks promising, where the definition of promising depends on the amount of knowledge gathered so far, guarantees the protection of the type I error rate. Also, in the many situations where the test statistic approximately follows a normal law, the deviation from the main result remains negligible. This article provides details regarding the Weibull and binomial distributions and indicates how one may approach these distributions within the current setting.

**Conclusions:**

There is thus reason to consider such designs more often, since they offer a means of adjusting an important design feature at little or no cost in terms of error rate.

## Background

The last few years interest in various adaptive trial designs has surged [1]. A greater flexibility of clinical study design and conduct has followed from the application of these new ideas [2]. In [1] adaptive designs are defined as: 

“...a clinical trial design that allows adaptations or modifications to aspects of the trial after its initiation without undermining the validity or integrity of the trial. ”

More and more trials of this sort are being reported and regulatory bodies take an increasingly favourable view on them [3]. All stand to win if these designs come to optimal use [4]. However, some concerns have been raised. One of these involve the risk of inflating type I error. The current article will assess that risk in the context of sample size adjustable (SSA) designs that allow choosing between raising the sample size, continuing as originally planned, or closing the trial due to futility.

The following will recapitulate parts of [5], add detail and draw conclusions for trial procedures. In that reference the authors show that if the interim results look promising, no inflation of type I error rate occurs. Here ’promising’ means that the conditional power at the current parameter estimate, i.e. the power updated by the accumulated knowledge at interim, amounts to at least 50%. This article will show that a less strict bound applies, in agreement with [6], exhibit the bound in terms of a test statistic, and present mathematical as well as computational aspects of it.

## Methods

### Assumptions

Denote the planned final sample size by *N*_0_, the number of patients available at the pre-planned interim analysis by *n*, and the possible raise determined at the interim taking conditional power into account by *r*. Let us consider a one-sided test at level *α* based on observing $X_{1},\ldots ,X_{N_{\text{final}}}$. Here *N*_*final*_ = *N*_0_ or *N*_*final*_ = *N* = *N*_0_ + *r*, depending on a decision taken during the course of the trial. The main result assumes normal distribution, but as will be outlined, it will still hold true for more general distributions. Further, assume the *X*_*i*_ to be independent normal with mean *θ* and variance 1. The null hypothesis states that *θ* = 0. Define the normalised test statistic *Z*^(*x*)^ by $Z^{\left(x\right)}=\sum_{i=1}^{x}X_{i}/\sqrt{x}$. The test rejects if $Z^{\left(N_{\text{final}}\right)}>z_{\alpha }$, where *z*_*α*_ is the 100 × (1 − *α*) percentile of the standard normal distribution: Φ(*z*_*α*_) = 1 − *α* (Φ being the cumulative distribution function of the standard normal distribution). The normalised test statistic $Z^{\left(n\right)}=\sum_{i=1}^{n}X_{i}/\sqrt{n}$ is observed when *n* patients have provided data, and the Data Monitoring Committee (DMC) will in part base its recommendations on the observed value. At this interim analysis an adaptation may lead to closing the study due to futility, continuing the study without changes or raising the sample size by recruiting an extra *r* subjects, yielding a total of *N* = *N*_0_ + *r* subjects. Closing the study due to futility may only decrease the type I error rate. So let us, for the sake of argument, disregard that possibility, and show that the type I error rate still remains protected.

The study protocol will specify *n* and *N*_0_, and at the interim we will consider raising the final sample size based on the conditional power evaluated at the current parameter estimate. Since the objective is to assess if the interim results are promising the current estimate of the parameter of interest gives the appropriate information [6]. As pointed out by Müller and Schäfer in [7], the over-all type I error can be preserved unconditionally under any general adaptive change, provided the conditional type I error that would have been obtained had there been no adaptation is preserved. This article however only considers the case of SSA. Unlike the situation in [8] the design does not permit sequential testing. Also, the article only considers the conventional hypothesis tests and p-values without adjustments.

We assess the conditional error rate as a function of *r*. By showing that the conditional type I error rate is bounded by the error rate which arises from the design without adaptation the unconditional error rate is proven to be controlled at a pre-specified level *α*.

### Derivation of the main result

We use the notation *X* ∼ *N*(*μ*,*σ*^2^) to signify that *X* follows a normal law with mean *μ* and variance *σ*^2^.

The change in type I error rate conditional on a sample size increase decided at the interim equals $G(r)=\text{Pr}(Z^{\left(N_{0}+r\right)}>z_{\alpha }|Z^{\left(n\right)}=z,\theta =0)-\text{Pr}(Z^{\left(N_{0}\right)}>z_{\alpha }|Z^{\left(n\right)}=z,\theta =0)$. The conditional distribution equals (*Z*^(*N*)^|*Z*^(*n*)^ = *z*,*θ* = 0)∼*N*(*ρz*, 1 − *ρ*^2^), where $\rho =\text{Cov}(\overset{N}{\underset{i=1}{\sum }}X_{i}/\sqrt{N},\overset{n}{\underset{j=1}{\sum }}X_{j}/\sqrt{n})=\overset{n}{\underset{i=1}{\sum }}1/\sqrt{\text{nN}}=\sqrt{n/N}$, and similarly for $Z^{\left(N_{0}\right)}$.

Expressing the difference in terms of normal distributions yields

$$G(r)=\Phi \left(\frac{\sqrt{\frac{n}{N_{0}+r}}z-z_{\alpha }}{\sqrt{1-\frac{n}{N_{0}+r}}}\right)-\Phi \left(\frac{\sqrt{\frac{n}{N_{0}}}z-z_{\alpha }}{\sqrt{1-\frac{n}{N_{0}}}}\right)$$

Now, in order to show this difference to be less than or equal to zero it may be equivalently shown that the difference of the arguments is negative (in the sense of non-positive), and denote this by *H*(*r*). Obviously *H*(0) = 0.

To simplify notation put *q* = *n*/(*N*_0_ + *r*) and *V* = (*N*_0_ + *r*)/*N*_0_ for arbitrary *n*,*N*_0_ and *r*, satisfying *N*_0_ > *n* > 0, and *r* > 0. Please note *qV* = *n*/*N*_0_. Then we aim to show

$$\frac{\sqrt{q}z-z_{\alpha }}{\sqrt{1-q}}-\frac{\sqrt{\text{qV}}z-z_{\alpha }}{\sqrt{1-\text{Vq}}}\leq 0$$

This implies

$$\;z\left(\frac{\sqrt{q}}{\sqrt{1-q}}-\frac{\sqrt{\text{qV}}}{\sqrt{1-\text{qV}}}\right)\leq z_{\alpha }\left(\frac{1}{\sqrt{1-q}}-\frac{1}{\sqrt{1-\text{qV}}}\right)$$

And, then, multiply by −1 and divide by the multiplier of *z*, to obtain

$$\;z\geq z_{\alpha }\frac{\sqrt{1-q}-\sqrt{1-\text{qV}}}{\sqrt{1-q}\sqrt{1-\text{qV}}}/)\frac{\sqrt{\text{qV}}\sqrt{1-q}-\sqrt{q}\sqrt{1-\text{qV}}}{\sqrt{1-q}\sqrt{1-\text{qV}}},$$

which after cancelling out $\sqrt{1-q}\sqrt{1-\text{qV}}$ becomes

(1) $$\begin{array}{ll} \;z & \geq z_{\alpha }(\sqrt{1-q}-\sqrt{1-\text{qV}})/(\sqrt{\text{qV}}\sqrt{1-q}-\sqrt{q}\sqrt{1-\text{qV}})\\ & :=z_{\alpha }b(q,V) \end{array}$$

Now let us compare this bound to $z_{\alpha }\sqrt{n/N_{0}}$. Denote the bound in (1) by *z*_*α*_*b*(*q*,*V*), and set out to prove $b(q,V)\leq \sqrt{\text{qV}}$. By subtracting $\sqrt{\text{qV}}$ from both sides and equating denominators we have

$$\;\frac{\sqrt{1\;-\;q}\;-\;\sqrt{1\;-\;\text{qV}}\;-\;\sqrt{\text{qV}}(\sqrt{\text{qV}}\sqrt{1\;-\;q}\;-\;\sqrt{q}\sqrt{1\;-\;\text{qV}})}{\sqrt{\text{qV}}\sqrt{1\;-\;q}\;-\;\sqrt{q}\sqrt{1\;-\;\text{qV}}}\;\leq \;0$$

But the denominator is positive under current assumptions. Thus, we may disregard it, and thus, we need to prove

$$\sqrt{1-q}(1-\text{qV})+\sqrt{1-\text{qV}}(q\sqrt{V}-1)\leq 0$$

Division by $\sqrt{1-\text{qV}}$, moving the right term to the right hand side of the inequality symbol, and squaring both sides yields

$$(1-q)(1-\text{qV})\leq 1-2q\sqrt{V}+q^{2}V$$

By expanding the left hand side product, eliminating terms and multiplying both sides by −1/*q*, we finally have

$$V+1\geq 2\sqrt{V},$$

which is true for all positive *V*.

Now regard *b* as a function of *r* for *n* and *N*_0_ fix. One may show that, $b(r)↗\sqrt{n/N_{0}}$ asymptotically as *r* ↘ 0. Further, *b* decreases as *r* grows in a close to linear fashion. Also, $b(r)↘(1-\sqrt{1-\text{qV}})/\sqrt{\text{qV}}$ when *r* tends to infinity.

Please note that since the $b(q,V)\leq \sqrt{\text{qV}}=\sqrt{n/N_{0}}$ a sufficient but not necessary condition is $z\geq z_{\alpha }\sqrt{n/N_{0}}$, which will be seen to give the conditional power 50% (the simple criterion). Consequently, this new criterion is less restrictive than the one presented in [5], and, importantly, changes with *r*. The reference [6] provides an example where the type I error remains intact although the conditional power descends down to 36%.

To obtain the conditional power please note that $E[Z^{\left(N_{0}\right)}|Z^{\left(n\right)}=z,\theta =z/\sqrt{n}]=z/\sqrt{n/N_{0}}$, and furthermore $\left(Z^{\left(N_{0}\right)}|Z^{\left(n\right)}=z,\theta =z/\sqrt{n})∼N(z/\sqrt{n/N_{0}},1-n/N_{0}\right)$. Then

$$\begin{array}{lll} \;\text{Pr}[Z^{\left(N_{0}\right)}>z_{\alpha }|Z^{\left(n\right)} & =z,\theta =z/\sqrt{n}]\; & \;\\ & =1-\Phi \left(\frac{z_{\alpha }-z/\sqrt{n/N_{0}}}{\sqrt{1-\frac{n}{N_{0}}}}\right)\; & \; \end{array}$$

The minimum of this probability over *z* > *b*(*q*,*V*)*z*_*α*_ equals

$$\Phi \left(\frac{z_{\alpha }(b(q,V)/\sqrt{\text{qV}}-1)}{\sqrt{1-\text{qV}}}\right)$$

From the definition of *G*(*r*) it follows that one cannot go further without increasing the conditional error rate. In this sense the bound is optimal.

### Weibull ditributed survival time points

We will now study the situation where survival times follow a Weibull distribution and right censoring time points are exponentially distributed.

In [9] the details of an Edgeworth expansion of the product limit estimator are given $\Phi (x)-n^{-\frac{1}{2}}\phi (x)(\tilde{\kappa_{3}}(x^{2}-1)+3\sigma_{1})/6$. First some notation: *X* = lifetime, *T* = left truncation time point, *Y*= right censoring time point, *Z* = *min*(*X*,*Y*), *δ* = *I*(*X* ≤ *Y*). Further, put *C*(*z*) := *P*(*T* ≤ *z* ≤ *Z*|*T* ≤ *Z*). But since *T* ≡ 0 this probability equals *P*(*Z* ≥ *z*) = *P*(*X* ≥ *z*,*Y* ≥ *z*) = *P*(*X* ≥ *z*)*P*(*Y* ≥ *z*). Then *W*_1_(*y*) := *P*(*Z* ≤ *y*,*δ* = 1) = *P*(*X* ≤ *y*,*Y* ≥ *X*). With $\sigma_{1}(z)=\int_{0}^{z}\frac{dW_{1}(u)}{C^{2}(u)}$, the constant $\tilde{\kappa_{3}}$ in the Edgeworth expansion equals $\sigma_{1}^{-1}\left(-7.5\sigma_{1}^{4}+\int_{0}^{z}C^{-3}(t)dW_{1}(t)\right)$. As stated we assume *X* ∼ *Weib*(*λ*,*β*) and *Y* ∼ *Exp*(*μ*). From this follows that *C*(*y*) = *exp*(−*μy*−*λy*^*β*^), $W_{1}(y)=\int_{0}^{y}e^{-\mu x}\lambda \beta x^{\beta -1}e^{-\lambda x^{\beta }}\text{dx}$. Thus one may at the interim use parameter estimates to calculate a normal approximation to the conditional power. Alternatively, one may simulate the remainder of the trial. A third option is to base the procedure on the logrank test whose statistic converges to a normal distribution. Consider the situation where the time to some event is compared between patients in an active treatment group and those in a control group. Let *r*_*i*_ refer to the number of patients remaining at time *i* and *o*_*i*_ refer to the number of observed events. Further, let *A* refer to the active treatment group and *C* to the control group. If $T=\overset{k}{\underset{i=1}{\sum }}\frac{r_{\text{iA}}o_{\text{iC}}-r_{\text{iC}}o_{\text{iA}}}{r_{i}}$ and $V=\overset{k}{\underset{i=1}{\sum }}\frac{o_{i}(r_{i}-o_{i})r_{\text{iA}}r_{\text{iC}}}{(r_{i}-1)r_{i}^{2}}$, then $z=T/\sqrt{V}$ will asymptotically be standard normal, e.g. [10]. Hence one may apply the simple criterion to *z* observed at the interim.

### Binomial proportion

For the sake of simplicity of exposition we focus attention to a single binomial proportion *p* and a one-sided test at the 5% level. Let the null hypothesis and alternative hypothesis be *H*_0_ : *p* = *p*_0_,*H*_1_ : *p* > *p*_0_. Please note that for $X_{N_{0}}∼\text{Bin}(N_{0},p_{0})$ the conditional distribution given {*X*_*n*_ = *k*} is the same as $X_{N_{0}-n}+k∼\text{Bin}(N_{0}-n,p_{0})$, and similarly for $X_{N_{0}+r}$.

From this follows that we may obtain *G*(*r*) exactly: in terms of *R* code [11]

However, we will look at a normal approximation. In the binomial case several test statistics with close to normal distribution exist: 

1. the score test statistic: $z=\sqrt{n}(\hat{p}-p_{0})/\sqrt{p_{0}(1-p_{0})})$,

$$\hat{p}=k/n$$

2. the log-odds: $z=\sqrt{n\hat{p}(1-\hat{p})}\left(\text{log}(\hat{p}/(1-\hat{p}))-\text{log}(p_{0}/(1-p_{0}))\right)$[12]

The simple criterion would then say that if *z* as above exceeds $\sqrt{\frac{n}{N_{0}}}z_{\alpha }$, then the procedures protects the type I error rate (unconditionally). But we set out to find a more accurate approximation.

Now using the condition {*X*_*n*_ = *k*}

$$\begin{array}{lll} \;G(r)= & P_{p_{0}}(X_{N_{0}+r-n}>q_{\alpha ,N_{0}+r}-k)\; & \;\\ & -P_{p_{0}}(X_{N_{0}-n}>q_{\alpha ,N_{0}}-k),\; & \; \end{array}$$

where *q*_*α*,*m*_ is the 100 × (1−*α*) percentile of *Bin*(*m*,*p*_0_).

Also the binomial distribution *X*_*n*_ ∼ *Bin*(*n*,*p*) admits a normal approximation of the pivotal statistic *U* = (*X*_*n*_ − *E*[*X*_*n*_])/*SD*(*X*_*n*_), which coincides with the score test statistic above, such that

$$P(U>q)=1-\Phi \left(q-\frac{1}{6\sqrt{n}}(\kappa_{3}(q^{2}-1))\right)+O\left(\frac{1}{n}\right),$$

in terms of the third cumulant of *U*, which picks up the skewness. As a rule of thumb it is often said that the normal approximation is quite accurate when *np* and *n*(1 − *p*) both exceed 5. But this statement holds even without the correction with respect to skewness.

In this case we may approximate the difference *G*(*r*) defined above by

$$\begin{array}{lll} \;G(r)= & P_{p_{0}}(X_{N_{0}+r}>q_{\alpha ,N_{0}+r}|X_{n}=k)\; & \;\\ & -P_{p_{0}}(X_{N_{0}}>q_{\alpha ,N_{0}}|X_{n}=k),\; & \; \end{array}$$

where we insert the percentiles obtained from the inversion of the Cornish-Fisher expansion, cf. e.g. [13]:

$$q_{\alpha ,n}=\mu_{n}+\sqrt{n}\sigma_{0}(z_{\alpha }+\frac{\gamma_{0}}{6\sqrt{n}}(z_{\alpha }^{2}-1)),$$

denoting *μ*_*n*_ = *np*_0_, $\sigma_{0}=\sqrt{p_{0}(1-p_{0})}$, and the third cumulant (1 − 2*p*_0_)/*σ*_0_ by *γ*_0_. This quantity will deviate less than 1 from the true percentile for n from 20 to 200, and *min*{*np*_0_,*n*(1 − *p*_0_)} > 5. Let us consider G(r) through the pivotal quantities

$$U_{N}=\frac{q_{\alpha ,N}-k-\mu_{N-n}}{\sigma_{N-n}}$$

We will be concerned with the difference

$$\begin{array}{lll} \;U_{N_{0}+r}-U_{N_{0}}= & \frac{q_{\alpha ,N_{0}+r}-k-\mu_{N_{0}+r-n}}{\sigma_{N_{0}+r-n}}\; & \;\\ & -\frac{q_{\alpha ,N_{0}}-k-\mu_{N_{0}-n}}{\sigma_{N_{0}-n}}\; & \; \end{array}$$

The task is now to identify when this difference is positive. To simplify notation denote by *n*_1_ the larger of the two sample sizes and by *n*_2_ the smaller. After equating the denominators and noting $\mu_{n_{i}}-\mu_{n_{i}-n}=np_{0},i=1,2$ the difference equals:

$$\frac{\sqrt{n_{2}-n}\left(np_{0}+\sqrt{n_{1}}z_{\alpha }\sigma_{0}+\gamma_{0}\sigma_{0}(z_{\alpha }^{2}-1)/6-k\right)}{\sqrt{\left(n_{1}-n)(n_{2}-n\right)}\sigma_{0}}$$

$$-\frac{\sqrt{n_{1}-n}\left(np_{0}+\sqrt{n_{2}}z_{\alpha }\sigma_{0}+\gamma_{0}\sigma_{0}(z_{\alpha }^{2}-1)/6-k\right)}{\sqrt{\left(n_{1}-n)(n_{2}-n\right)}\sigma_{0}}$$

Disregarding the positive denominator yields the condition:

$$\begin{array}{ll} \left(\sqrt{n_{2}-n}-\sqrt{n_{1}-n})(np_{0}+\frac{\gamma_{0}\sigma_{0}(z_{\alpha }^{2}-1)}{6}-k\right) & \;\\ \;+(\sqrt{n_{2}-n}\sqrt{n_{1}}-\sqrt{n_{1}-n}\sqrt{n_{2}})z_{\alpha }\sigma_{0}>0 & \; \end{array}$$

Some algebra will unearth the condition

$$\begin{array}{lll} \;k> & np_{0}+\frac{\gamma_{0}\sigma_{0}(z_{\alpha }^{2}-1)}{6}\; & \;\\ & \;\;\;\;+\frac{(\sqrt{n_{2}-n}\sqrt{n_{1}}-\sqrt{n_{1}-n}\sqrt{n_{2}})z_{\alpha }\sigma_{0}}{\sqrt{n_{2}-n}-\sqrt{n_{1}-n}}\; & \; \end{array}$$

Please note that the first term corresponds to expectation of the null distribution. Further, the second term will be negative if *p*_0_ > 0.5, and the third will always be negative under the conditions of this paper. From this follows that the normal approximation of *G*(*r*) is non-positive for *k* satisfying the above condition. Finally, invoke the fast convergence of the binomial distribution towards a normal law, which means that already 20 observations will make the normal approximation quite accurate, provided *min*{*np*,*n*(1 − *p*)} > 5. Simulations indicate that this decision rule is accurate already at an interim sample size *n* as low as 20. However, the rule does not guarantee preservation of the conditional type I error rate for all *p*. Thus the conclusion is that for the binomial distribution there is no inflation of the unconditional type I error rate under the above conditions. A total of 900000 simulations with *n* from 20 to 100, *p*_0_ picked randomly in [5/*n*,1−5/*n*], *k* randomly generated from *Bin*(*n*,*p*_0_) and *N*_0_ = 2*n* and *r* = *n* gave a median and mean of *G*(*r*) equal to −0.004762 and −0.004574, respectively, over the set defined by the inequality above. A set of similar simulations using the simple criterion ( $k>n(p_{0}+\sqrt{p_{0}(1-p_{0}))/N_{0}}z_{\alpha }$) gave median and mean equal to −0.02429 and −0.02389, respectively. Thus the simple criterion will be on the conservative side.

## Results

### Main result

In **Methods** the following result was derived.

#### A conditional power that quarantees preservation of nominal significance level

If the conditional power at the interim, which occurs after *n* out *N*_0_ planned observations and leads to a raise of *r*, equals at least

(2) $$\Phi \left(\frac{z_{\alpha }(b(q,V)/\sqrt{\text{qV}}-1)}{\sqrt{1-\text{qV}}}\right),$$

where *q* = *n*/(*N*_0_ + *r*), *V* = (*N*_0_ + *r*)/*N*_0_, and

(3) $$\;b(q,V)=(\sqrt{1\;-\;q}\;-\;\sqrt{1\;-\;\text{qV}})/)(\sqrt{\text{qV}}\sqrt{1\;-\;q}\;-\;\sqrt{q}\sqrt{1\;-\;\text{qV}}),$$

then the type I error rate is preserved. The function *b* satisfies the inequalities $(1-\sqrt{1-\text{qV}})/\sqrt{\text{qV}}\leq b(q,V)\leq \sqrt{\text{qV}}=\sqrt{n/N_{0}}$.

A more practical criterion, or rule of thumb, may be to derive a test statistic *z* with close to a standard normal distribution under the null hypothesis, and check whether $z>\sqrt{\frac{n}{N_{0}}}z_{\alpha }$. This will be referred to as the simple criterion, and stems from [5]. More generally, the condition *z* > *b*(*q*,*V*)*z*_*α*_ suffices (cf. equations (11) and (12) in [6]). The conditional power bound in (2) decreases as *r* increases, but the lower bound on *b* implies a limit.

### Example

Take the example of *n* = 55,*N*_0_ = 110,*r* = 40 and *α* = 0.025,*z*_*α*_ = 1.96. Then the minimum conditional power equals 43%, see next subsection. Thus a conditional power of considerably less than 50% is permissible from the point of view of type I error rate preservation. This may be good to know if the original sample size calculation was grossly wrong. Then recruiting more subjects than planned may resolve the issue without jeopardising the type I error rate. On the other hand, in such a situation the validity of the scientific hypotheses on which the trial design rests may be questioned, and the sponsor will have to judge whether the updated hypotheses suggest a commercially viable route. Nevertheless, in some cases raising the sample size will make sense, and may save the trial from unnecessary disaster.

Above we assume the variance to be known. If it is not we may estimate it and use for instance a *t*-test statistic which quickly converges to a normal as the sample size increases.

Examination of the *t*-test has provided evidence of a small degree of inflation [14]. In [15] further details of when inflation occurs are given. However, already at a sample size of 30 the *t*-distribution and the normal distribution appear almost identical.

### Calculations in R

In the statistical software environment R [11] one may easily define functions. Let us regard the bound *z*_*α*_*b*(*q*,*V*) as a function of (*n*,*N*_0_,*r*) instead. We may explore the bound *z*_*α*_*b*(*n*,*N*_0_,*r*) through the R function *B.func*

and, the minimum conditional power through

So, for instance *CP.min(alpha1 = 0.025, n = 55, N0 = 110, r = 40)* = 0.43, and, *B.func(n = 55, N0 = 110, r = 0.01)* = 0.7070907, which approximately equals $\sqrt{n/N_{0}}=\sqrt{55/110}\approx 0.7071068$. Also *CP.min(alpha1 = 0.025, n = 55, N0 = 110, r = 110)* = 0.3575873.

### Deviations from normal distribution

If we use non-normal data such as survival type of data, then it is often possible to approximate the test statistic by a normal variate. Many test statistics, e.g. those derived by the maximum likelihood method, converge quickly to a normal distribution when the sample size increases. This feature extends the relevance of the main result to measurements following other distributions than the normal.

In **Methods** we looked into the situation where a Kaplan-Meier (KM) estimate is used. The Edgeworth expansion of the distribution of the (standardised) KM estimator has the form $\Phi (x)-n^{-\frac{1}{2}}\phi (x)\tilde{\kappa_{3}}(x^{2}-1)/6$, where $\tilde{\kappa_{3}}$ is specified in **Methods**[9,16], Φ the cumulative distribution function of a standard normal variate and *ϕ* its frequency function. So if we express the change in conditional error rate (*G*(*r*) below) in terms of this expansion the correction term to difference between normal distribution functions will approach zero as $1/\sqrt{n}$. Assuming some parametric distribution, such as the Weibull distribution, one may work out the details regarding this approximation. Or, one may assess the deviation from normality through a simulation procedure.

In the case of a single binomial proportion *p* and a one-sided test of the null hypothesis *H*_0_ : *p* = *p*_0_ versus the alternative hypothesis *p* > *p*_0_, it holds that if we at the interim observe *X*_*n*_ = *k* satisfying

$$\begin{array}{lll} \;k> & np_{0}+\frac{\gamma_{0}\sigma_{0}(z_{\alpha }^{2}-1)}{6}\; & \;\\ & \;\;\;\;+\frac{(\sqrt{N_{0}-n}\sqrt{n_{1}}-\sqrt{n_{1}-n}\sqrt{N_{0}})z_{\alpha }\sigma_{0}}{\sqrt{N_{0}-n}-\sqrt{n_{1}-n}},\; & \; \end{array}$$

with *σ*_0_ = *p*_0_ (1−*p*_0_), *γ*_0_ = (1−2*p*_0_)/*σ*_0_ and *n*_1_ = *N*_0_ + *r*, then inflation of the type I error rate will not occur. More precisely put: on average, over all possible outcomes, the procedure will preserve the type I error rate. However, the conditional error rate will not always fall below the nominal one.

## Discussion

There are operational issues with adaptive designs that must be addressed during the planning stage. In order to safeguard the integrity of the trial and avoid operational bias following an unblinded interim precautions need to be put in place to limit access to both the results and, even, the analysis plans. The latter will specify the output and decision rules, but will leave open the possibility of including other information, such as external factors in the final decision whether to stop for futility or to continue, and if so, whether or not to raise the sample size.

Further, a number of concerns have been raised involving the risk of violating statistical principles or lack of efficacy compared to group sequential designs, e.g. [17-19].

However valid these objections may be, more and more practitioners have felt that the challenges are tractable and have found SSA designs an attractive option. For small biotechnology companies this option gives the possibility of starting a trial with rather limited resources, followed by an additional investment conditional on the interim results being promising. Also, the SSA design makes a lot of sense whenever a fix size design would have to rely on quite limited amount of information regarding the primary variable.

Several references have argued the superiority of seamless phase II/III designs over the traditional phase II and III trials. Merging the two phases produces gains in valuable time [20], and, under reasonable conditions, saves sample size [21].

Earlier research has established that a conditional power at the interim analysis exceeding 50% implies that the conditional, and hence also the unconditional, type I error rate is preserved, cf. [5,7]. Further, the reference [6] builds on [8] and others to identify a more general region where this happens. The region is identified through equations (11) and (12) in [6]. The derivation of the region relies on results for Brownian motion. Together these two equations implicitly define a bound that coincides with *b* in (3) above.

Further, one cannot use a lower bound without risking inflation of the conditional error rate, and thus one may not rely on the Müller-Schäfer principle of conditional error functions [7] (new does not exceed the original) to prove preservation of unconditional error rate^1^. By virtue of the Müller-Schäfer principle of conditional error functions any interim decision rule, pre-defined or not, that does not violate this fundamental requirement will permit a redesign of the trial. So from this perspective the SSA designs described here are well behaved and offer great flexibility.

## Conclusions

This article has shown that the risk of compromising the nominal significance level of a statistical test by allowing a sample size increase during the course of a trial remains low and controllable. The conditional error rate and power provide key decision tools.

## Endnotes

^1^ Also, by reversing the order of terms in *G*(*r*) and tracing the same line of thought one may conclude that a sample size decrease is permissible when results are discouraging. But then it may make more sense to discontinue the trial due to futility.

## Competing interests

The author declares that he has no competing interests.

## Pre-publication history

The pre-publication history for this paper can be accessed here:

http://www.biomedcentral.com/1471-2288/13/94/prepub
